# Microbial Transcription Factor-Based Biosensors: Innovations from Design to Applications in Synthetic Biology

**DOI:** 10.3390/bios15040221

**Published:** 2025-03-31

**Authors:** Kyeongseok Song, Haekang Ji, Jiwon Lee, Youngdae Yoon

**Affiliations:** Department of Environmental Health Science, Konkuk University, Seoul 05029, Republic of Korea; kssong1999@konkuk.ac.kr (K.S.); wl08140@konkuk.ac.kr (H.J.);

**Keywords:** transcription factors (TFs)-based biosensors, genetic engineering, dynamic metabolic control, high-throughput screening (HTS), strain evolution

## Abstract

Transcription factor-based biosensors (TFBs) are powerful tools in microbial biosensor applications, enabling dynamic control of metabolic pathways, real-time monitoring of intracellular metabolites, and high-throughput screening (HTS) for strain engineering. These systems use transcription factors (TFs) to convert metabolite concentrations into quantifiable outputs, enabling precise regulation of metabolic fluxes and biosynthetic efficiency in microbial cell factories. Recent advancements in TFB, including improved sensitivity, specificity, and dynamic range, have broadened their applications in synthetic biology and industrial biotechnology. Computational tools such as Cello have further revolutionized TFB design, enabling in silico optimization and construction of complex genetic circuits for integrating multiple signals and achieving precise gene regulation. This review explores innovations in TFB systems for microbial biosensors, their role in metabolic engineering and adaptive evolution, and their future integration with artificial intelligence and advanced screening technologies to overcome critical challenges in synthetic biology and industrial bioproduction.

## 1. Introduction

Over the past few decades, biosensors have been extensively studied and applied across various industrial fields to detect and monitor specific targets [1,2,3,4]. In general, biosensors consist of sensing elements that recognize targets and signal-transducing elements that convert these responses into measurable outputs [5,6]. Biosensors were initially devised as alternative tools for monitoring hazardous materials arising from rapid industrial growth that pose significant threats to human health. Consequently, various biosensors based on DNA, antibodies, fluorescent proteins, enzymes, and aptamers have been developed and further diversified by integrating signal-transducing devices such as microfluidics, surface plasmon resonance (SPR), electrodes, and surface-enhanced Raman scattering (SERS) [7,8,9,10]. Advances in nanotechnology and materials science have further accelerated biosensor development by integrating these innovations into sensor components [11,12,13].

Depending on the integrated biomolecules, biosensors are classified into several categories, including nanomaterial-, nucleic acid-, and transcription factor (TF)-based biosensors [14,15,16]. Each type of biosensor has distinct advantages and disadvantages depending on the nature of the biomolecules employed. For instance, nucleic acid-based biosensors excel in generating target specificity; however, the stability of nucleic acids is a critical limitation. Similarly, biosensors employing proteins face challenges related to the stability and regeneration of the active proteins. However, the primary goal of biosensors is to quantitatively determine targets with high accuracy and sensitivity.

Although the primary focus of biosensors has been to rapidly and precisely monitor targets, recent studies have expanded their scope of application. Among the various biosensors, genetically encoded biosensors such as transcription factor (TF)-based biosensors (TFBs) and riboswitches have gained attention owing to their ability to activate and regulate the expression levels of reporter genes in response to target concentrations [16,17,18]. These systems differ in their regulatory elements, with TFs in TFBs being protein-based and riboswitches being RNA-based *cis*-acting regulators. As their working mechanisms rely on the transcription and translation of genes through interactions with specific targets, they have been extensively applied in synthetic biology to construct genetic circuits [19,20,21,22]. Although both systems show promise as gene-regulating tools, TFBs are more actively applied in diverse research fields because of their inherent advantages, such as greater stability, reduced susceptibility to environmental factors, and improved structural predictability compared with nucleic acid-based components such as aptamers, ribozymes, and riboswitches [21,23]. Compared with traditional biosensors that rely on enzymes, antibodies, or synthetic nanomaterials, TFBs offer several unique advantages. These include real-time intracellular sensing, genetic tunability, modular design, and seamless integration with host regulatory networks. Such characteristics position TFBs as central components in the development of next-generation biosensor technologies, particularly in synthetic biology and metabolic engineering.

The unique characteristics of TFBs, specifically their ability to regulate gene expression in response to signals, with expression levels varying according to signal concentrations, have facilitated their application across various fields. With the accumulation of knowledge regarding TFs and their corresponding targets, numerous TFBs have been developed and employed to quantify targets in diverse research domains. For instance, TFs such as ArsR, ZntR, MerR, and MntR have been used to detect and monitor heavy metals in environmental systems [24,25,26]. Over time, the scope of TFB targets has expanded to include cellular metabolites driven by the increasing availability of genetic and functional data on TFs across various organisms [27,28,29]. Recently, TFB applications have expanded to include high-throughput screening, adaptive evolution, metabolic pathway engineering, and target monitoring, driven by recent advancements in TFs and TFB systems [30,31,32]. However, the performance of native TFB systems remains limited because of their relatively weak specificity, broad target selectivity, and constrained dynamic ranges [29,31,32].

Despite the promising aspects of TFB systems, their limitations must be addressed to fully realize their potential. As noted in previous studies, TFs often exhibit broad selectivity and specificity toward targets. Because these characteristics directly influence the TFB performance, modulating the selectivity and specificity of TFs is critical for the development of effective TFB systems. Significant efforts have been made to improve TFB performance through the genetic engineering of TFs [33,34]. Moreover, the dynamic range of TFBs—the fold change in gene expression between the presence and absence of inducers—is another crucial factor affecting the translational output of target genes. Achieving optimal dynamic ranges requires fine-tuning TFs, promoters, operators, and ribosome-binding sites using engineering approaches [35,36,37].

In this review, we summarize the fundamental principles of TF-based biosensor systems and highlight the recent advances in this field. We also explore strategies to improve TFB performance and expand their application areas. Finally, we discuss the latest trends in TFB applications and the future potential of these systems across various research and industrial domains. To establish the context for TF-based biosensors, it is important to first understand the broader category of genetically encoded biosensors. The following section introduces the main types and sensing mechanisms of genetically encoded biosensors, which form the conceptual foundation for TF-based biosensor development.

## 2. Overview of Genetically Encoded Biosensors

Genetically encoded biosensors are generally classified based on their working mechanisms as allosteric TF-based biosensors, nucleic acid-based biosensors, and fluorescent protein-based biosensors. Nucleic acid-based biosensors utilize nucleic acids such as aptamers and riboswitches as sensing elements in conjunction with reporter genes. Compared with other genetically encoded biosensors, nucleic acid-based biosensors offer superior target sensitivity and selectivity. Additionally, rapid advancements in (systematic evolution of ligands using exponential enrichment) SELEX have made it easier to obtain aptamers tailored for detecting specific targets [38,39]. However, a major limitation is that the functionality of aptamers in vitro often cannot be replicated effectively in vivo [40]. Fluorescent protein-based biosensors detect various metabolites by measuring fluorescence activities, such as bimolecular fluorescence complementation (BiFC) or fluorescence resonance energy transfer (FRET) signals, which are induced by ligand interactions [41,42]. Although these biosensors are effective for sensing, their inability to regulate the downstream gene translation limits their application in metabolic engineering compared with other types of biosensors. In contrast, TF-based biosensors can regulate gene expression in response to specific target concentrations, making them particularly suitable for metabolic engineering applications [21,43]. Although the specificity and selectivity of TFs are generally weaker than those of aptamers, the sensing elements in nucleic acid-based biosensors can be enhanced through the genetic engineering of TFs and their operating DNA sequences. Therefore, TF-based biosensor systems are considered to have significant application potential compared with other types of genetically encoded biosensors.

Among the various genetically encoded biosensors, transcription factor-based biosensors (TFBs) have attracted particular attention due to their ability to directly regulate gene expression in response to intracellular signals. Their inherent advantages—such as modularity, stability, and tunability—make them powerful tools in metabolic engineering and synthetic biology. The next section delves into the fundamental components and working principles of TF-based biosensor systems, outlining how they detect signals and generate outputs through engineered regulatory elements.

## 3. TF-Based Biosensor Systems

### 3.1. The Mechanisms of Action

**Figure 1:**
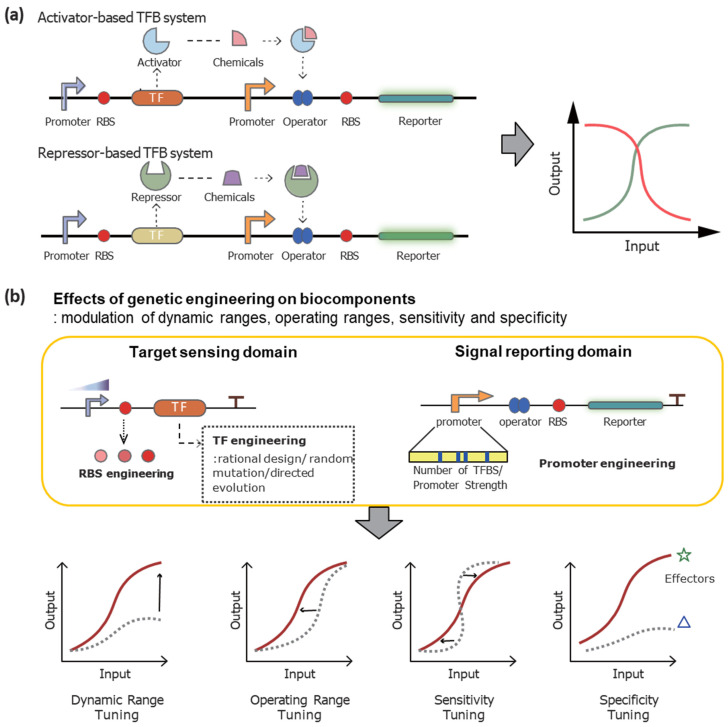
Working mechanisms of TFB systems and the effects of biocomponents. (**a**) Working mechanisms of activator-based and repressor-based TFB systems. The interaction between TFs and targets affects the expression of reporter genes, and it was translated as output signals. (**b**) Effects of genetic engineering of biocomponents, including promoter, RBS, TFBS, operator, and TFs, on the performances of TFB systems.

Diverse chemical sensors and biosensors operate on the same fundamental principle: the sensing component recognizes targets, this recognition induces output signals, and the signal is then transduced into digitized values. TFBs function by utilizing TFs to detect specific analytes and regulate gene expression, thereby linking biological signals to measurable outputs. Its mechanism of action involves three main steps: analyte recognition, signal transduction, and output generation. As shown in Figure 1a, TFs bind to target molecules, inducing conformational changes that either activate or repress their interaction with specific promoter regions. This interaction influences the expression of downstream reporter genes, producing detectable signals such as fluorescence, luminescence, or colorimetric changes. Depending on the nature of TFs, reporter gene expression may be either activated or repressed in the presence of their targets.

For example, MerR family TFs, such as MerR and ArsR, respond to heavy metals, such as mercury and arsenic, by modulating the expression of genes linked to luminescent reporters, enabling environmental monitoring [44,45]. In addition, there are many other TFs responsive to heavy metals, such as MntR for manganese, ZntR for cadmium and zinc, PbrR for lead, and ChrB for chromate, and they have been introduced as sensing elements in biosensor systems for monitoring the corresponding heavy metals [24,25]. Similarly, LuxR, a quorum-sensing TF that recognizes acyl-homoserine lactones (AHLs), has been used as a sensing element in TF-based biosensors, making it a critical tool for the detection of microbial infections in the clinical field [46,47]. Moreover, TFs that respond to antibiotics, cellular metabolites, and chemicals have been identified and used as genetic systems in biosensors [21,48,49]. As TFB systems control the expression of downstream genes, they have been considered building blocks (modules) to construct synthetic pathways, as well as tools to monitor harmful materials and pathogens. For instance, a MerR-based biosensor was engineered to detect mercury ions in environmental water samples, achieving a detection limit of 0.2 μg/L. This system enables real-time monitoring of heavy metal contamination, demonstrating its field applicability for environmental risk assessment.

Table 1 summarizes the TFs for the aforementioned target-monitoring TFB systems and recent studies with these characteristics. These TFB systems highlight the adaptability and impact of TFBs across the environmental, clinical, and industrial domains, driving innovation in monitoring, diagnostics, and production systems. Continuous advancements in engineering and computational approaches will further enhance the functionality and scope of biosensor applications.

### 3.2. Engineering Strategies

Although TF-based biosensor systems hold greater potential for expanding their applications, their limitations, such as relatively weak specificity, broad target selectivity, and constrained dynamic ranges, must be improved to fully realize their potential [29,31,32]. As discussed in previous sections, the performance of TFBs is influenced by biocomponents such as TFs, promoters, enhancers, and RBSs (Figure 1b). Additionally, the choice of reporter genes and host strains significantly impacts the characteristics of TFB systems. Because the combined effects of these factors determine overall TFB performance, they have become key targets for genetic engineering. The following sections explore engineering strategies aimed at optimizing these factors to enhance TFB performance. A summary of these engineering strategies is provided in Table 2.

#### 3.2.1. Genetic Engineering on TFs to Modulate TFB Systems

**Figure 2:**
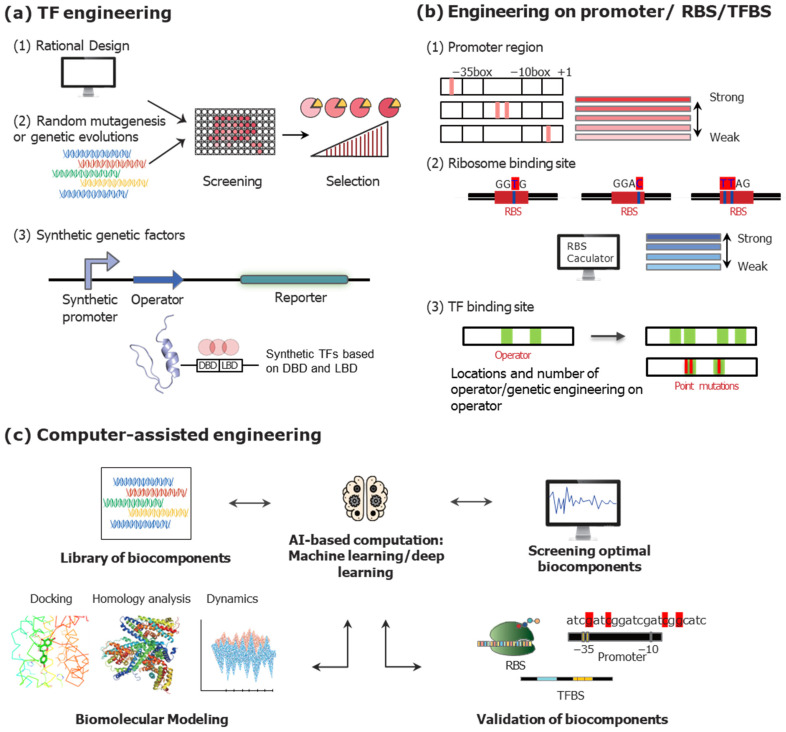
Genetic engineering strategies to modulate the performance of TFB systems. (**a**) The strategies to modulate the characteristics of TFs by genetic engineering. (**b**) Genetic engineering on promoter, RBS, and TFBS. (**c**) Computer-assisted engineering on biocomponents.

In general, the target specificity and sensitivity of TFBs are determined by the intrinsic properties of TFs. The interaction between TFs and their targets governs target selectivity and sensitivity, which, in turn, influences the dynamic range and detection limit of TFBs. Therefore, the genetic engineering of TFs through rational design or random mutagenesis has emerged as a key strategy for modulating the performance of TFBs (Figure 2a).

As an example, rational mutagenesis of the ZntR regulator led to the development of a variant with enhanced selectivity for cadmium over zinc. The engineered TFB system exhibited a 3-fold improvement in sensitivity and a narrower detection window, enabling more accurate quantification of Cd(II) in mixed-metal samples [65,66]. Furthermore, engineering ZntR in combination with the deletion of the ion channel responsible for heavy metal export in *E. coli* significantly enhanced sensitivity by disrupting the homeostasis system, thereby increasing intracellular target accumulation [81]. Similarly, Machado et al. employed a PcaV repressor to construct protocatechuic acid (PCA) biosensors and subsequently generated vanillin biosensors through the directed evolution of PcaV. They obtained a PcaV mutant named Van2 and constructed a vanillin-specific TFB system [67].

Studies have demonstrated that engineering TFs to modulate TFB properties has been actively pursued, unlocking the potential of TFB systems for diverse applications. With the rapid advancement in new technologies, TF engineering has expanded beyond rational design and random mutagenesis to include computer-assisted design. Computer-aided protein engineering, previously used to enhance catalytic activity and antibody development, has also been extended to TF engineering [82,83]. Advances in mathematical algorithms, including deep learning and ML, have further enabled the prediction of TFB system performance based on extensive datasets [84,85,86]. Moreover, numerous studies have demonstrated the engineering of TFs to refine and enhance TFB system performance [16,87].

#### 3.2.2. Engineering on DNA Sequences to Optimize TFB Systems

The performance of TFBs depends on various factors that influence target recognition and signal production. In TFB systems, target recognition is typically indicated by reporter gene expression. Engineering DNA sequences, such as promoters, operators, ribosome binding sites (RBS), and transcription factor binding sites (TFBS), plays a pivotal role in optimizing the performance of TFB systems. Additionally, the sensitivity, selectivity, and dynamic range of TFB systems are influenced by the expression levels of both TFs and reporter genes, which can be fine-tuned through genetic engineering or optimization.

For example, Chen et al. optimized promoter sequences in an ArsR/P_ars_-based biosensor system, achieving an improved detection limit for arsenic from 50 ppb to 9.38 ppb and broadening the dynamic range. These modifications demonstrate that engineering regulatory elements can substantially enhance the practical performance of TFB systems [71]. Xu et al. developed a malonyl-CoA-sensing *E. coli* strain using a *fap*O repressor and the FapR transcription factor [73]. They constructed regulatory architectures by combining the T7 promoter and *lac*O into a TFB based on FapR/*fap*O, allowing the system to be regulated by two inducers. This dual regulation enabled precise control of the dynamic range and optimized carbon flux for the biosynthesis of malonyl-CoA-derived compounds. Similarly, Liang et al. designed an artificial regulatory circuit using the HucR repressor and the P_hucR_ promoter by engineering both TFs and promoters [74]. They screened HucRs and their corresponding promoters from a genetic library and demonstrated that the sensitivity, dynamic range, and response time of biosensors can be fine-tuned by engineering components. Through this multi-layered dynamic control, they enhanced the vanillin production efficiency, achieving a 27-fold induction for the HucR variant and an approximately 10-fold increase in vanillin production for the P_hucR_ variant. In another study, Dabirian et al. proposed a strategy to regulate the dynamic range of *fapO*/FapR-based biosensors by modifying the position and number of TF-binding sites in the promoter region [35]. By inserting binding sites at different positions within the constitutive promoter P*_CCW12_*, they modulated the dynamic range of the TFB system from 95-fold to 2.4-fold. Furthermore, the presence of multiple repressors, such as FapR and Mig1, in hybrid promoter systems influences the dynamic range of TFB systems. Genetic engineering strategies targeting the promoters, RBSs, and TFBSs discussed above are illustrated in Figure 2b.

Building on these approaches, researchers have increasingly utilized synthetic or hybrid promoters to enhance TFB performance. Synthetic promoters have been designed to fine-tune the expression of target genes in various microorganisms, providing greater flexibility and control over synthetic pathways [88,89]. Advances in computational techniques, particularly artificial intelligence (AI), have accelerated the design and construction of synthetic promoters by enabling precise prediction of promoter activity [90,91]. Although computational approaches for designing synthetic promoters are not the primary focus of this review, their applications in AI-assisted rational design and activity prediction for optimizing TFB systems will be discussed later.

In conclusion, genetic engineering and DNA sequence optimization determined the expression levels of regulatory proteins and reporter genes, which directly influenced the characteristics of TFB systems. Therefore, targeting the DNA sequences through engineering is a critical strategy for enhancing the performance of TFB systems and addressing their limitations.

#### 3.2.3. Recent Trends of Engineering Strategies to Enhance the TFB Systems

While the advantageous aspects of TFB systems highlight their potential for diverse research applications, several limitations arising from the intrinsic properties of transcription factors (TFs)—such as broad selectivity, weak specificity, and a limited repertoire—must be addressed to realize their full potential. Beyond the approaches discussed earlier, novel approaches have been developed and applied to enhance TFB system performance (Figure 2). In the following sections, the strategies to enhance the TFB systems shown in Figure 2 were discussed. Besides these approaches, recent advances in synthetic biology and genome engineering have introduced powerful tools to address these challenges. For example, CRISPR-Cas9 and dCas9-based systems have enabled the construction of programmable transcriptional circuits, allowing precise modulation of gene expression levels [22,92,93]. Synthetic transcription regulators and modular promoter libraries have further expanded the design space for customizable TFBs [94,95]. Although they were not discussed in this review, these innovations enable dynamic control, enhanced responsiveness, and broader applicability of biosensors in both research and industrial contexts.

##### Design of Synthetic TFs

One major challenge is the limited number of available TFs compared with the vast array of possible targets, including harmful substances and metabolic compounds. Although engineering native TFs can expand their target range, this alone is insufficient for addressing this problem. To overcome these limitations, synthetic transcription factors (sTFs) were developed to regulate and fine-tune gene expression. These sTFs are typically constructed by combining DNA-binding and effector domains, thereby enabling the regulation of gene expression in response to specific DNA sequences [96,97]. Khalil et al. introduced a synthetic framework for constructing eukaryotic transcription functions using artificial zinc fingers (ZFs) as the modular DNA-binding domains [76]. They demonstrated the use of ZFs as transcriptional activators by regulating *yEGFP* expression downstream of the ZF operators. Their study revealed that synthetic TF promoter pairs generated from ZF libraries and operator sequences exhibited diverse activation levels. They further evaluated the effects of the promoter/operator number and the level of ZF-DNA binding affinity on activation, proposing that subtle perturbations in DNA-TF interactions, promoter design, and specificity can significantly influence the signal-processing behavior of TFB systems. Similarly, Chen et al. developed a TFB system for *S*-Adenosylmethionine (SAM) using a synthetic TF comprising an *E. coli*-derived DNA-binding domain (MetJ), a human estrogen receptor-binding domain (hER), and a viral activation domain VP16 [77]. Although TFB systems employing sTFs remain underexplored, the rapid advancement of technology, particularly in AI, is likely to make sTFs a critical component of future TFB systems.

##### Computer-Assisted Engineering Strategies

Computer-assisted engineering has gained prominence in the field of TFB systems because of its ability to identify transcription factors (TFs) and their binding sites and to predict the activity and dynamic ranges of TFB systems. The performance of TFB systems depends on the combined action of TFs, their binding sites, inducers, promoter strength, and expression levels. While traditional rational design approaches may not always achieve the desired performance, computer-assisted methods leveraging deep learning or machine learning offer a solution by utilizing extensive databases accumulated over decades to enable the design of TFB systems with predictable performance [98,99,100]. These methods also significantly reduce the time and labor associated with traditional approaches and enhance precision by predicting the activity of key biocomponents, such as promoters, TFBS, RBS, and TFs, making AI-based rational design a highly effective strategy [86,101]. Recognizing the immense potential of artificial intelligence (AI), researchers have developed various algorithms to extract and utilize large datasets for specific applications. AI-based models have been applied to the rational design of biocomponents to improve the performance of TFB systems [80,102,103,104]. Although this review does not focus on the development of AI models or algorithms, we highlight recent studies showing their application in enhancing TFB system performance. For example, Ding et al. developed an AI model called CLM-RDR, a classification model based on deep learning between cross-RBSs (cRBSs) and the average dynamic range, and elucidated the performance of CLM-RDR on glucarate, arabinose, and glycolate biosensors with 72.2%, 61.1%, and 50% prediction accuracy rates, respectively [75]. Similarly, Zhou et al. reported ML algorithms to predict dose-response relationships based on a dataset obtained from a trackable combinatorial library containing 5184 combinations of TF dosage, operator positions, and upstream enhancer sequences (UAS) [79]. They used a malonyl-CoA biosensor employing FapR-f*ap*O as a model system and obtained the biosensor with the largest dynamic response range. Additionally, they suggested that the pipeline provides an efficient, affordable, and universal platform that enables high-throughput screening of dose-response curves and facilitates the rational design of genetic circuits. Moreover, a deep-learning model called DeepSTARR was developed by Almeida et al. to predict enhancer activity [80]. Recent advancements in transcription factor (TF) motif identification and higher-order regulatory syntax have enabled the de novo design of synthetic enhancers with tunable activity, as demonstrated by tools like DeepSTARR. While these enhancer designs were not originally developed for TF-based (TFB) biosensor systems, they can be readily integrated into such platforms, potentially enhancing system performance. To fully realize this potential, it is essential to validate the combined effects of promoters, enhancers, and ribosome binding site (RBS) sequences using AI-driven models.

Despite progress in using AI to decipher the relationships between genetic elements and gene expression, the targeted design of biocomponents specifically for TFB systems remains relatively underexplored. However, the incorporation of machine learning (ML) into biosensor development is gaining momentum. ML models have been successfully applied to predict TF-binding specificity, optimize genetic circuitry, and inform the construction of synthetic regulatory networks. Notably, transformer-based architectures and tools such as DeepTFactor have shown strong capabilities in modeling TF-promoter interactions [105,106].

Overall, the integration of computational design with refined sensing elements and modular genetic components has significantly advanced TFB biosensor systems. These innovations have expanded their applicability, enabling robust use in high-throughput screening, strain engineering, and metabolic pathway optimization, establishing their value in both synthetic biology research and industrial biotechnology.

## 4. Applications of TF-Based Biosensor Systems

In recent years, TFB systems have been employed for the detection and monitoring of a wide range of analytes, including environmental pollutants, pathogens, diseases, and cellular metabolites, across various industries. These systems rely on the specificity of transcription factors (TFs) to recognize target analytes, and as a result, the performance and specificity of TFB systems are fundamentally determined by their associated TFs. Numerous TFs capable of interacting with analytes, such as heavy metals and toxic chemicals, have been identified and applied in TFB systems. The application areas of TFB systems are not confined to analyte detection but vary depending on the type of analyte being targeted. For example, they are increasingly used in synthetic biology for natural product biosynthesis, improving microbial cell factory performance for substance production, and facilitating adaptive evolution to select highly efficient strains. The following discussion focuses on these broader applications beyond analyte detection, with examples listed in Table 3.

### 4.1. TFB Systems on High-Throughput Screening

HTS is a method used to select strains with the highest efficiency for the desired performance through fast and simple processes. As mentioned earlier, several critical factors, such as promoters, TFs, and enhancers, play significant roles in enhancing the performance of TFB systems. Unlike rational design, random genomic mutagenesis of these factors can generate extensive libraries that require the identification of the desired mutations and strains. However, this process is often tedious, time-consuming, and labor-intensive. These challenges can be addressed by employing HTS techniques that integrate microfluidics with fluorescence-activated cell sorting (FACS), Raman-activated cell sorting, and mass spectrometry, leveraging recent advancements to maximize throughput [124]. One notable example is the use of a TF-based biosensor for erythromycin detection, developed by Wang et al. Utilizing the MphR transcription factor and RBS engineering, the authors established a screening platform that enabled efficient identification of high-yield strains from a large library. As a result, erythromycin production was improved by 6.8-fold compared with the parent strain. This case illustrates the direct impact of TFB systems in accelerating the strain optimization process and reducing time and cost in industrial antibiotic production pipelines [107]. Binder et al. employed a LysG-based biosensor to achieve enhanced lysine production, with titers reaching 68.3 g/L, representing a 21% improvement over parental strains [108]. Similarly, genetically encoded biosensors based on the camphor-responsive TetR family regulator CamR from *Pseudomonas putida* have been used for high-throughput screening and dynamic regulation of bicyclic monoterpene-producing strains [109]. In addition, there are many other studies linking TFB systems to HTS, which could be a tool for screening positive strains obtained from the directed or adaptive evolution of strains.

### 4.2. TFB Systems on Stain Evolution

Directed and adaptive evolution are widely employed strategies for optimizing biological systems by introducing genetic variation and subsequently selecting desirable traits [125,126,127]. Both approaches rely on iterative cycles of mutation and selection to enhance functionality. While conceptually distinct, these methods share a common objective and often require the use of high-throughput screening (HTS) strategies, such as fluorescence-activated cell sorting (FACS) and microfluidic-based fluorescence-activated droplet sorting (FADS) [87,91]. In this context, transcription factor-based (TFB) systems play a pivotal role in strain evolution by transducing cellular metabolite signals into growth-selectable phenotypes or reporter signals suitable for HTS.

Leavitt et al. demonstrated the use of adaptive laboratory evolution (ALE) coupled with synthetic biosensors to enhance the production of muconic acid in *Saccharomyces cerevisiae* [112]. Their approach combined ALE with rational metabolic engineering and a biosensor module responsive to endogenous aromatic amino acids (AAAs) as a proxy for pathway flux [128]. This strategy resulted in a yeast strain capable of producing 0.5 g/L of muconic acid in shaking flasks, the highest titer reported to date for *S. cerevisiae* [112]. Similarly, Chen et al. developed an *E. coli* strain with the highest efficiency of myrcene production through the directed evolution of myrcene synthase. Their HTS strategy employed a TFB system based on the MyrR and *P_myr_* pair to select the highest-titer producers [113], and Dietrich et al. achieved a 120-fold enrichment in 1-butanol production strains using a TFB system incorporating BmoR-P_BMO_ [114]. Another compelling example of practical application comes from the work of Siedler et al., who employed a QdoR-based biosensor to screen for enhanced flavonoid-producing strains. By applying this system to a mutant library, they successfully identified high-producing variants of flavonol synthase (FLS1), resulting in a kaempferol yield of 56 mM per OD600 in *E. coli* [60]. This demonstrates not only the effectiveness of TFB systems in adaptive evolution but also their potential in natural product discovery and pharmaceutical biosynthesis. Tong et al. recently demonstrated the use of a TtgR/*ttgO*-based TFB system to enhance naringenin production [115]. These examples highlight the potential of TFB systems to facilitate strain evolution by enabling the selection of desired traits. In addition, Chou et al. demonstrated the application of feedback-regulated evolution of a phenotype (FREP), which is an adaptive control system designed to dynamically modulate mutation rates. This system increases the mutation rate to generate genetic diversity and decreases it as the concentration of a target metabolite increases, thereby maintaining the selection pressure [116]. Using FREP, they evolved *E. coli* strains with enhanced production of tyrosine and isopentenyl pyrophosphate (IPP) and isolated the evolved strains by monitoring fluorescent protein levels. Although this approach introduced mutations during strain evolution, it reinforced the critical role of TFB systems in strain development. In addition to the aforementioned studies, several different TFB systems have been reported and applied to strain evolution over the past few decades.

In conclusion, TFB systems have emerged as powerful tools for strain evolution, enabling precise selection of desirable traits through integration with advanced high-throughput screening methods. Despite some limitations, such as the dependency on the availability of suitable biosensors and the potential introduction of unintended mutations, TFB systems hold significant promise for future applications. As these systems continue to evolve and improve, their use in adaptive and directed evolution strategies is likely to expand, driving advancements in metabolic engineering and bioproduction efficiency.

### 4.3. Metabolic Engineering for Synthetic Biology

Metabolic engineering seeks to maximize the production of target metabolites by precisely redirecting biosynthetic pathways while minimizing the negative impacts on cellular growth [27,129,130]. Achieving this balance requires reducing the energy burden on cells, preventing the accumulation of metabolic intermediates, and maintaining an equilibrium between cell growth and metabolite biosynthesis [117,131]. Metabolic engineering is pivotal for enhancing the production yield and biosynthetic efficiency. However, significant challenges remain, including the identification of optimal strains from large libraries, implementation of rational designs, and effective redirection of metabolic pathways. TFB systems address these limitations by providing real-time feedback mechanisms to regulate gene expression based on the target metabolite concentrations, thereby advancing metabolic engineering strategies. Numerous studies and reviews have highlighted the application of TFB systems in metabolic engineering and microbial cell factories, highlighting their ability to improve productivity and streamline biosynthetic processes [27,130,132]. The roles of TFB systems in metabolic engineering are aligned with their core functionalities of sensing target metabolite concentrations and regulating genes. Researchers have adapted TFB systems to diverse applications, demonstrating their flexibility and efficacy. Recent findings further reinforce their potential, as elaborated in the following sections.

Zhang et al. developed a dynamic sensor-regulator system (DSRS) to enhance fatty-acid-based product synthesis in *Escherichia coli* and demonstrated its application in biodiesel production [117]. Using the FadR/P*_fadBA_* system, they constructed fatty acid/acyl-CoA biosensors to identify strains with the highest biodiesel production capabilities. This study highlights the potential of DSRS strategies to optimize biosynthetic pathways by balancing metabolic fluxes, thereby increasing product titers, improving conversion yields, and stabilizing host production. These findings underscore the versatility and potential of TFB systems in advancing metabolic engineering. Similarly, the feedback-regulated evolution of phenotypes (FREP) exemplifies the application of TFB systems to enhance metabolite production [116]. Moreover, TFB systems have proven to be versatile tools for dynamic metabolic pathway regulation and reconstruction of genetic circuits [21,132,133]. For instance, Zhu et al. developed bifunctional glycolysis biosensors based on the Cra transcription factor, which functions as both an activator and repressor, depending on the promoter context. This system enabled the dynamic control of glycolysis flux in *E. coli*, resulting in a strain capable of producing 111.3 g/L mevalonate without generating by-products [118]. They introduced synthetic promoters of varying strengths or phage-derived promoters into this system to generate a biosensor for detecting fructose-1,6-diphosphate (FBP). This TFB system improves pyruvate and lycopene production in *E. coli* by regulating the ATP synthesis and membrane synthesis genes *plcC*, respectively [119].

Xu et al. constructed a bifunctional genetic circuit incorporating a pyruvate-responsive biosensor using PdhR to regulate glucaric acid synthesis [120]. By dynamically regulating the *ino1* gene and inhibiting glycolysis and the pentose phosphate pathway, they achieved a 2.5-fold increase in glucaric acid production, which was further improved by 4-fold by suppressing by-product formation. In a complementary approach, Verma et al. proposed a method to quantify performance trade-offs in metabolite biosensor designs [121]. By optimizing the flux-versus-burden trade-off, the efficiency of metabolic engineering can be enhanced, demonstrating an approach for improved glucaric acid production in *E. coli*.

However, these studies predominantly focused on single TFB systems for the dynamic control of synthetic pathways despite the fact that target metabolites are often synthesized through multiple interconnected metabolic pathways. To address this limitation, researchers have focused on integrating TFB systems into multi-layered genetic circuits. For example, Moon et al. introduced the concept of applying logic gate designs to biological systems [122], and Xiang et al. proposed constructing metabolic networks capable of integrating multiple signals, activating genes, and producing the desired outputs [134]. These efforts have culminated in the development of Cello, a computational tool that enables the in silico design of genetic circuits. Notably, Cello successfully designed 60 genetic circuits for *E. coli*, 45 of which performed as expected, thereby producing the desired output [123].

Collectively, these studies highlighted the critical role of precise regulation in balancing cell growth and biosynthetic efficiency to maximize metabolite production in synthetic biology. TFB systems not only enable tight control of biosynthetic pathways to prevent precursor depletion but also integrate seamlessly with computational tools such as Cello for in silico design of complex genetic circuits. By coordinating cellular growth, metabolite synthesis, and multi-layered genetic networks, TFB systems are driving innovations in metabolic engineering, paving the way for scalable and efficient production of high-value compounds.

As TF-based biosensor systems continue to evolve in terms of design and functionality, their impact on synthetic biology, metabolic engineering, and microbial production is becoming increasingly profound. Despite the remarkable progress made, several challenges remain that must be addressed to fully harness their potential. The following concluding section summarizes the key insights from this review and discusses future directions for expanding the utility and performance of TF-based biosensors in both academic and industrial contexts.

## 5. Conclusions and Prospectives

TFBs have been established as transformative tools in diverse fields, including metabolic engineering, synthetic biology, and environmental monitoring [21,30,113,132]. Their capacity to transduce intracellular metabolite concentrations into quantifiable outputs is indispensable for the dynamic regulation of metabolic pathways, high-throughput screening (HTS), and strain evolution. This review explores the recent advancements in TFB systems, underscoring their applications in microbial production optimization, synthetic pathway construction, and adaptive and directed evolution strategies. Engineering efforts have substantially improved the specificity, sensitivity, and dynamic range of TFBs, broadening their applicability to complex biological systems and industrial-scale processes [74,85,127].

Despite these advances, several challenges must be addressed to fully realize the potential of TFB systems. A key limitation is the restricted repertoire of transcription factors capable of recognizing a broad range of metabolites, which limits the applicability of the TFB system. To address this challenge, genome mining and synthetic biology approaches, including the rational design of artificial TFs, offer promising avenues for expanding the diversity of available biosensors [27,88,90]. Additionally, improving the robustness of TFB systems to endure industrial stress conditions is essential for their successful implementation in large-scale bioproduction and process optimization.

The future integration of TFB systems with emerging technologies such as artificial intelligence (AI) and machine learning (ML) presents immense opportunities [80,83,98]. AI-driven methodologies can streamline the rational engineering of TFs, promoters, and ribosome-binding sites, thereby optimizing TFB performance for specific metabolic and biotechnological applications. The combination of TFB systems with advanced HTS technologies, including microfluidic droplet sorting and Raman-activated cell sorting, has accelerated the discovery and selection of high-performing microbial strains. In synthetic biology, TFB systems are expected to evolve into modular components for constructing sophisticated genetic circuits and feedback-regulated networks, enabling precise control over cellular functions.

In conclusion, TFB systems have emerged as a cornerstone of modern biosensor technologies, providing a versatile platform for integrating metabolic sensing with dynamic regulatory control. Their continued development, driven by innovations in genetic engineering, computational tools, and synthetic biology, will likely expand their utility in addressing pressing challenges in biotechnology, medicine, and environmental science. By bridging the gap between metabolic sensing and precise regulatory control, TFB systems play a pivotal role in redefining the frontier of microbial and synthetic biology.

## Figures and Tables

**Table 1:** Examples of TFs employed TFB systems for target monitoring.

	Targets	TFs	Origin	Dynamic Range and DL	Ref.
Heavy metals	Hg(II)	MerR	*E. coli* *P. luminescens*	0.78–12.5 μM; 0.39 μM 0.4–1600 μg/L; 0.2 µg/L	[50] [51]
	Cu(II)	CueR	*E. coli*	0.39–78.68 μM	[52]
	As(III), As(V)	ArsR	*E. coli*	10 µg/L	[53]
	Zn(II), Hg(II), Cd(II)	ZntR	*E. coli*	3–30, 30–300, 0.01–1 μM	[24]
	Pb(II)	PbrR	*C. metallidurans*	0.2 to 0.05 μg/mL	[54]
	Mn(II)	MntR	*E. coli*	0.01–10 µM	[25]
Organic chemicals	3-HBA Tetracycline	MobR TetR	*C. testosterone* *E. coli*	2 mM 1.25 μM	[55]
	3-MBz	BenR	*E. coli*	0.1–1.0 mM	[56]
	Salicylic acid	MarR	*E. coli*	5 µM	[57]
	tetracycline	TetR	*E. coli*	0.05–0.15 µM	[58]
	TCDD	AhR-ARNT	human	10 fM	[59]
Flavonoids	Kaempferol Quercetin	QdoR	*E. coli*	0.01–0.05 mM 0.01–0.05 mM	[60]
	Naringenin	FdeR	*E. coli*	0.01–0.05 mM	[60]
	Phloretin Genistein	TtgR	*E. coli*	0.01–0.1 mM 0.001–0.1 mM	[58]
Quorum sensing molecules	HSLs and AHLs	LasR QscR LuxR RhlR	*P. aeruginosa* *P. aeruginosa* *V. fischeri* *P. aeruginosa*	pM—μM 0.01–0.1 μM - -	[61] [62] [63]
	Autoinducer-2	LuxR	*V. harveyi BB170*	0.25 pM	[64]

3-HBA, 3-hydroxybenzoate; 3-MBz, 3-methylbenzoate; TCDD, 2,3,7,8-tetrachlorodibenzo-*p*-dioxin; AhR, aryl hydrocarbon receptor; ARNT, AhR nuclear translocator (ARNT); HSLs, homoserine lactone; AHLs, N-acyl homoserine lactone.

**Table 2:** Examples of engineering strategies to modulate performances of TFB systems.

	Genetic System	Strategies	Effects on Performances of TFB Systems	Ref.
TF engineering	ZntR-P_zntA_	Replacing the MBLs Rational design-based mutagenesis on ZntR	Broad specificity of TFB modulated to Hg and Cd specific Enhancing Cd and Hg sensitivity	[65] [66]
	PcaV-P_PV_	Direct evolution on PcaV	Selectivity shifted from PCA to vanillin	[67]
	MarR-*marO*	Rational design-based mutagenesis on MarR	Modulating the specificity and selectivity of TFB system to aspirin	[48]
	PocR-P_cob_	Mutation on PocR to modulate the interaction with activator	Interaction with activator altered the level of RNA polymerase recruiting, regulating sensitivity, and dynamic ranges of TFB system	[68]
	AraC	Direct evolution of AraC and TetA-based dual-selection by introducing OA as a ‘decoy’ ligand	Improvement of selectivity and sensitivity of TFB systems toward ligands about 24-fold compared with native TFB systems	[69]
	LacI	LacI engineering by saturation mutagenesis	Altering effector specificity to lactulose and applied to C2E evolution to enhance lactulose production	[70]
Engineering on DNA sequences	ArsR-P_ars_	Promoter sequence optimization and TFBS adjustment	Enhancing the sensitivity (9.38 ppb of DL) and expansion of the dynamic range (0–5 ppb)	[71]
	MarR- *marO*	Modulating the strength of promoters	Modulating the dynamic ranges of TFB system toward SA	[72]
	FapR-*fap*O FapR	Insertion of *lacO* between promoter and *fapO* Modification and re-localization of TFBS on promoter	Enhancing biosynthesis of malonyl-CoA-derived compounds by controlling the dynamic range and optimized carbon flux The dynamic ranges of TFB system modulated by types of promoters and the number of TFBS	[73] [35]
	HucR-P_hucR_	Mutation on HucR and modifying promoter sequences	Increase sensitivity to vanillin about 27-fold and 10-fold increase in vanillin production by engineering	[74]
	CdaR	Library screening of RBSn and RBSm for TFs and reporter genes	Evaluating the effects of combining both RBSs and constructing powerful platform to tune the dynamic range of biosensors by deep learning	[75]
Synthetic TFs	ZFs-synthetic operators	Construction of TFB systems based on various combinations of ZF-based TFs and operators	TFB systems showed different dynamic ranges upon the sequences of sTF and operators; the outputs were modulated by genetic components	[76]
	MetJ-hER-VP16	Construction of sTF by conjugating MetJ, hER, and VP16	TFB system based on synthetic TF responds to SAM in a dose-dependent manner	[77]
	Acla-P_AraC_	Replacing the LBD of AraC with IsoA to construct chimeric TF	Modulating sensitivity and specificity of TFB system toward isoprene by employing chimeric TF	[78]
Computer-assisted engineering	RBS	Construction of CLM-RDR by deep learning of large datasets cRBSs	AI-based RBSs design and verifying the prediction accuracy using arabinose and glycolate biosensors	[75]
	Enhancer/ Operator	Construction of MLalgorithm to predict dose-response relationship	Prediction the genotype-phenotype relationships based on biocomponents	[79]
	Enhancer	a deep learning model, DeepSTARR, to predict activity of enhancers	de novo design and functional validation of synthetic enhancers with desired activities	[80]

MBLs, metal-binding loops; PCA, protocatechuic acid; OA, orsellinic acid; C2E, cellobiose 2-epimerase; TFBS, transcription factor binding site; RBS, ribosome binding site; sTF, synthetic TF; ZFs, zinc fingers; hER, human estrogen receptor binding domain; SAM, *S*-Adenosylmethionine; Acla, chimeric AraC-IsoA; LBD, ligand-binding domain; cRBS, cross-RBS; ML, machine learning.

**Table 3:** Recent studies on applications of TF-based biosensor systems.

	Targets	TFs	Origin	Roles of TFB Systems	Outcome	Ref.
HTS	erythromycin	MphR	*S. erythraea*	Screening strain libraries of RBS engineering	A total of 6.8-fold increase in erythromycin production	[107]
	Lysine	LysG	*C. glutamicum*	Screening strain from library generated by MNNG treatment	A total of 21% improvement in lysine production	[108]
	BMP	CamR	*P. putida*	Promoter, operator, and RBS engineering and CamR evolution	increased the system’s signal-to-noise ratio to 150-fold.	[109]
	D-allulose	PsiR	*A. tumefaciens*	Selecting RhaD mutants from directed evolution	Two superior strains isolated from 40,000 colonies	[110]
	GA	CdaR	*E. coil*	Selecting high GA-producing strain	A total of 17-fold increase in GA production	[111]
Directed/adapted evolution	AAA	ARO80	*S. cerevisiae*	High AAA-producing strain selection from ALE	the highest MA-producing titer reported to date	[112]
	*β*-myrcene	MyrR	*Pseudomonas* sp.	Applied for directed evolution of myrcene synthase	the highest titer reported to date: 510.38 mg/L of myrcene	[113]
	1-butanol	BmoR	*P. butanovora*	Selection of high 1-butanal-producing strain	A total of 120-fold enrichment for a 1-butanol	[114]
	Kaempf	QdoR	*B. subtills*	Selection of high kaempferol-producing strains from library	A total of 56 mM of kaempferol produced per OD_600_ in *E. coli*	[60]
	Naringenin	TtgR	*P. putida*	Selecting enhanced *CHS* from directed evolution	increasing the naringenin titer by 65.34%	[115]
	IPP	sTF	*E. coli*	Selection of high IPP-producing strains induced by *mutD*5	Increase the IPP production by *E. coli* evolved by FREP	[116]
Metabolic engineering	Fatty acid	FadR	*E. coli*	Regulation of FAEE production pathway by the DSRS	A total of 1.5 g/L and 3-fold yield increase in FAEE	[117]
	FBP	Cra	*E. coli*	Dynamic control of glycolysis flux in *E. coli*	A total of 111.3 g/L of mevalonate without generating by-products	[118]
	FBP	Cra	*E. coli*	Dynamic control of the following: (1) Target ATP Synthesis Gene (2) Membrane Synthesis Gene	Increasing production of the following: (1) Pyruvate (9.66 g/L) (2) Lycopene (100.3 mg/L)	[119]
	pyruvate	PdhR	*B. subtills*	Design genetic circuits for dynamic dual control (activation and inhibition)	Four-fold increase in glucaric acid production	[120]
Programming genetic circuits	Model to optimize performance trade-off in the design of metabolite biosensors			Optimizing the flux-versus-burden trade-off	Design a kinetic model for dynamic control circuits	[121]
	Ara, IPTG, aTC, and etc.	AraC, TetR, Laci, Sica, InvF, and etc.		Transducing the input signals to layering logic gates	Construction of logic gates and a design strategy for integrated circuits	[122]
	Computational tool, Cello, to construct in silico design for genetic circuits			A genetic module to regulate input and output signals	Forty-five out of sixty designed circuits for *E. coli* performed	[123]

MNNG: N-methyl-N’-nitro-N-nitrosoguanidine; BMP: bicyclic monoterpene; GA: glucaric acid; AAA: aromatic amino acid; ALE: adaptive laboratory evolution; Kaempf: kaempferol; MA: muconic acid; *CHS*: chalcone synthase; IPP: isopentenyl pyrophosphate; FREP: feedback-regulated evolution of phenotype; FAEE: fatty acid ethyl ester; DSRS: dynamic sensor-regulator system; FBP: fructose-1,6-diphosphate; Ara: arabinose; aTC: anhydrotetracycline.

## Data Availability

Not applicable.
